# The Effect of Graphene Oxide Deposition, Shot Peening, and Hybrid Graphening on the Structural and Mechanical Properties of 30HGSA Steel

**DOI:** 10.3390/ma18214853

**Published:** 2025-10-23

**Authors:** Sebastian Stabryn, Barbara Nasiłowska, Robert Szczepaniak, Mateusz Mucha, Grzegorz Mońka, Tomasz Rygier, Wojciech Chrzanowski, Maciej Chrunik, Piotr Olejnik, Marta Kutwin, Zdzisław Bogdanowicz

**Affiliations:** 1 Faculty of Aviation, Polish Air Force University, Dywizjonu 303 no. 35, 08-521 Deblin, Poland; s.stabryn@law.mil.pl (S.S.); m.mucha@law.mil.pl (M.M.); 2 Institute of Optoelectronics, Military University of Technology, gen. S. Kaliskiego 2, 00-908 Warsaw, Poland; 3 Łukasiewicz Research Network—Warsaw Institute of Technology, Duchnicka 3, 01-976 Warsaw, Poland; grzegorz.monka@wit.lukasiewicz.gov.pl (G.M.); tomasz.rygier@wit.lukasiewicz.gov.pl (T.R.); 4 Faculty of Materials Science and Engineering, Warsaw University of Technology, Wołoska 141, 02-507 Warsaw, Poland; 5 Faculty of Mechanical Engineering, Military University of Technology, gen. S. Kaliskiego 2, 00-908 Warsaw, Poland; wojciech.chrzanowski@wat.edu.pl (W.C.); zdzislaw.bogdanowicz@wat.edu.pl (Z.B.); 6 Institute of Applied Physics, Military University of Technology, gen. S. Kaliskiego 2, 00-908 Warsaw, Poland; maciej.chrunik@wat.edu.pl; 7 Department of Physics and Biophysics, Warsaw University of Life Sciences (SGGW), 159 Nowoursynowska Street, 02776 Warsaw, Poland; piotr_olejnik@sggw.edu.pl; 8 Department of Nanobiotechnology, Institute of Biology, Warsaw University of Life Sciences, Ciszewskiego 8, 02-786 Warsaw, Poland; marta_kutwin@sggw.edu.pl

**Keywords:** 30HGSA, graphene oxide, shot peening, hybrid graphening

## Abstract

This publication presents the performance properties of 30HGSA steel after various surface treatments involving hybrid graphene coating, shot peening, and graphene oxide coating, and of the material in its delivery state used in aerospace structures. Performance tests were carried out on the structure, measuring surface roughness, microhardness, corrosion, residual stresses and bending strength for all surface treatments. It has been demonstrated that hybrid graphitization results in increased surface roughness, increased compressive stress and a beneficial increase in the bending strength of the sample compared to other research groups. A new method of strengthening steel surfaces by hybrid graphitization, consisting of coating the steel surface with graphene oxide and shot peening, has been described. The mechanism of hybrid graphitization affecting the increase in the performance properties of 30HGSA steel, including a 43% increase in maximum bending strength compared to BM, has been presented.

## 1. Introduction

Due to the dynamic loads, they are subjected to during operation, the materials used in aeronautical structures must have optimal functional properties [1,2,3,4,5]. The technological challenge is to develop structural materials characterized not only by low weight but also by corrosion resistance and high mechanical strength. One method of increasing fatigue life is surface shot peening. The results of research conducted by Nakonieczny [6] and Nasiłowska [7] showed that the impact of individual shot particles on the material surface leads to a number of beneficial effects, including an increase in microhardness and the induction of compressive stresses.

Testing of aluminum metal samples subjected to shot peening for 30, 60, 90, 120 and 150 s showed that shot peening for up to 90 s significantly increases microhardness and surface roughness. After this time, the obtained parameters become stable. A further increase in microhardness occurs, but only slightly [8]. When selecting shot peening parameters, the type of shot material must be considered. It should have a similar elemental composition to the workpiece material to prevent the transfer of foreign elements that may act as corrosion initiators.

Xie et al. [9] conducted research on the surface modification of Ti-15V-3Cr-3Al-3Sn using shot peening, followed by the application of an epoxy resin combined with graphene oxide to the surface layer. Shot peening, performed prior to the application of the anti-corrosion coating, was shown to enhance both corrosion resistance and coating adhesion. The introduction of graphene nanostructures into graphene-based coatings to improve corrosion properties has been the subject of many studies [7,9,10]. These studies, although conducted at independent research centres, showed that the best corrosion resistance was achieved with a coating containing approximately 0.1 wt.% graphene nanostructures. The addition of 0.1 wt.% graphene oxide (GO) to a low-viscosity epoxy matrix improved the dispersion of GO, increasing the adhesion of the coating to the substrate, which consequently had a positive effect on the anticorrosion performance in NaCl electrolyte.

In the study by [9], the surface shot peening process of Ti-15-3-3-3 was performed in a wet environment to increase surface roughness. In the subsequent stage, the treated material was immersed in an epoxy resin containing graphene oxide (GO). The samples prepared in this manner were subjected to salt spray corrosion resistance testing.

The use of graphene and its derivatives in the form of graphene oxide as a nanocomposite coating was investigated by Amrollahi et al. [11]. A significant improvement was achieved not only in corrosion resistance, including under atmospheric conditions, but also in coating adhesion strength.

Composites based on 7050 aluminum alloy reinforced with graphene nanoparticles were the subject of studies by Venkatesan et al. [12]. It was observed that the best material properties were obtained with the addition of 0.3 wt.% graphene, whose presence was a decisive factor influencing the improvement of tensile strength.

Ward et al. [4] carried out an analysis of plasma-modified coatings intended to improve the functional properties of engine components in the aerospace industry. These coatings were reinforced with multilayer graphene oxide (GO). It was shown that the addition of GO to the NiAl coating exhibited high potential for various regenerative and operational applications, while increasing hardness, adhesion, and fatigue resistance by 49%, 18%, and 14%, respectively. The enhancement of mechanical properties resulted from the appropriate orientation of GO structures during solidification, improved platelet adhesion at reinforcement interfaces, and a reduced fraction of undesired oxide phases in the microstructure [13].

Although numerous scientific publications demonstrate the corrosion-resistance imparted by graphene nanostructures deposited on steel surfaces [14,15,16,17,18,19], and others show that shot peening enhances mechanical properties [6,7,20,21,22,23,24,25,26], combining these two surface-layer improvement techniques still poses a challenge that calls for detailed investigation. To advance the current state of knowledge, this paper investigates the influence of hybrid graphening on the residual stresses and mechanical properties of 30HGSA steel. The publication describes a new surface treatment method in the form of hybrid graphitization combining graphene oxide deposition and surface shot peening. This method has not been described in publications to date, which represents an entry into a new area of research involving the introduction of graphene nanostructures into surface layers for the purpose of surface reinforcement. In addition to the author’s method [27], the publication also introduces new terminology for *hybrid graphening*.

## 2. Materials and Methods

Samples with the dimensions specified in Table 1 were cut from a 30HGSA steel (VIGRAMET, Koło, Poland) sheet measuring 2000 × 1000 × 3 mm using a water jet. The shape of the samples depended on the research methodology adopted and the limitations of the equipment used.

The edges of the specimens were machined using a milling machine. Four test groups of specimens were analyzed according to the following designations:

- BM: 30HGSA steel in the base material;
- BM + GO: 30HGSA steel with a deposited graphene oxide layer;
- BM + SP: 30HGSA steel after surface shot peening;
- BM + GO + SP: 30HGSA steel with a deposited graphene oxide layer that was then subjected to surface shot peening.

This process is referred to as hybrid graphening (according to patent [27]).

### 2.1. Object of Research

#### 2.1.1. 30HGSA Steel

According to material certificate No. 3966-16b, the elemental composition of 30HGSA steel is shown in Table 1. The certificate also states that a 30HGSA steel sheet measuring 1000 × 2000 × 3 mm was heat-treated (oil quenching at 890 °C followed by tempering at 540 °C) to achieve a hardness of 207 HB. Additionally, the manufacturer specified yield and tensile strengths of 910 MPa and 1160 MPa, respectively. The sheets were cleaned by sandblasting in the delivery condition.

All tests were conducted on samples measuring 70 × 20 × 3 mm, except for the metallographic test.

#### 2.1.2. Dispersed Aqueous Suspension of Graphene Oxide

A dispersed aqueous suspension of graphene oxide at a concentration of 4.5 g/L was purchased from the Chemical Synthesis and Flake Graphene Department of the Łukasiewicz Research Network—Institute of Electronic Materials Technology in Warsaw. The graphene oxide (GO) flakes in the suspension were approximately 1.0–1.5 nm thick and 3–10 µm in size, with an oxygen content of 45–52%. The GO flakes in the suspension were produced using the Hummer method [28].

### 2.2. Sample Preparation Methodology

#### 2.2.1. Methodology for Surface Deposition of Graphene Oxide Layer

To ensure permanent deposition of the graphene oxide (GO) layer and its incorporation into the surface layer, a plasma cleaning process was performed prior to GO deposition using a Plasma Prep III device generating 100 W RF plasma. The use of plasma cleaning increases the hydrophilicity of the steel surface, allowing the aqueous GO suspension to wet the surface more effectively. This promotes uniform distribution of graphene oxide flakes contained in the dispersed suspension across the entire surface and facilitates the adhesive integration of GO particles into the steel’s surface layer [7].

Immediately after removal from the plasma chamber, the samples were placed in a Petri dish containing an aqueous graphene oxide suspension with a concentration of 4.5 g/L. After 15 min, the samples were removed and the excess suspension was mechanically removed. GO layer deposition was applied to the BM + GO and BM + GO + SP specimen groups.

#### 2.2.2. Surface Shot Peening Methodology

**Figure 1:**
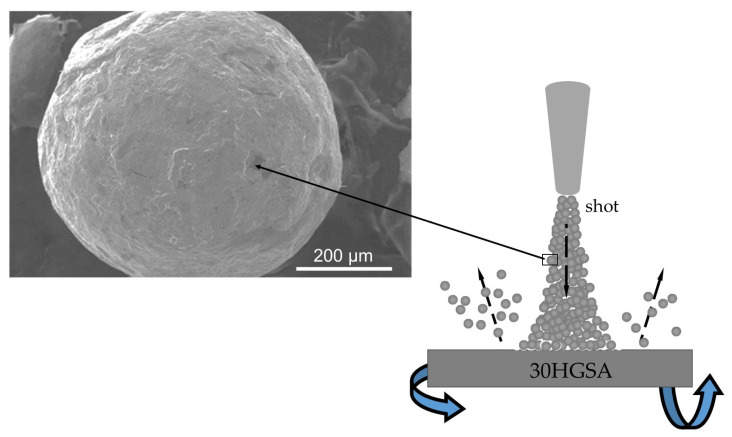
Surface shot peening diagram.

The surface layer strengthening process was carried out at a shot peening station, type PEEN–IMP [29], located at the Institute of Precision Mechanics in Warsaw (currently: Łukasiewicz Research Network—Warsaw Institute of Technology). The system allows for continuous adjustment of the impact energy of the shot stream. The shot, which was in the shape of balls, was directed perpendicularly to the specimen, which was rotating during the process (Figure 1).

The following shot peening parameters were selected: austenitic stainless steel shot (C~0.07%, Cr~18%, Ni~10%) with diameters of 0.2 mm and 0.3 mm; a pressure of *p* = 5 bar, and exposure time of 15 min. The peening intensity, as measured using Almen plates type N, was fN = 0.24 mm, with 100% surface coverage. The distance between the nozzle assembly and the peened surface was l = 430 mm.

#### 2.2.3. Hybrid Graphening Process Methodology

**Figure 2:**
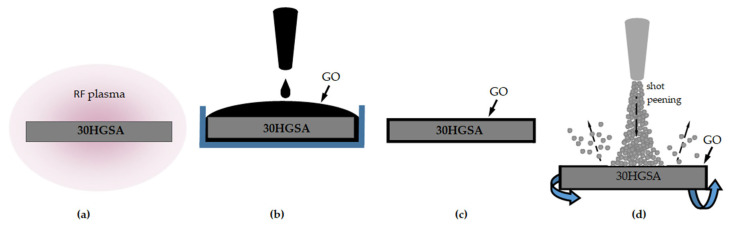
Schematic of the specimen strengthening process via hybrid graphening, i.e., surface cleaning and activation using plasma (**a**), deposition of graphene oxide (**b**,**c**), and shot peening of the surface coated with the graphene oxide layer (**d**) [7].

The hybrid graphening process consists of the following sequential stages:

(1) Surface cleaning and activation using low-frequency RF plasma (Figure 2a).

(2) Deposition of graphene oxide (Figure 2b,c).

(3) Shot peening of the surface coated with the graphene oxide layer (Figure 2d) [7]. Cleaning the 30HGSA steel surface using plasma took 15 min at a power of 100 W (SPI Supplies Plasma Prep III Etcher, Solid State Design) (Figure 2a). Immediately after removal from the RF plasma reaction chamber, the samples were placed in a beaker containing 4.5 g/L^3^ for 15 min (Figure 2b). The excess suspension was then removed mechanically (Figure 2c), after which the samples were placed in a Vacucell 22 L vacuum dryer (BMT Medical Technology s.r.o., Brno-Zábrdovice, Czech Republic). In the final stage, the surface was shot peening according to the parameters specified in the relevant Section 2.2.2.

### 2.3. Research Methodology

#### 2.3.1. Structural Analysis

The graphene oxide suspension was applied to TEM grids intended for transmission imaging using (Ted Pella, Inc., Po Boy Ln, Cottonwood, CA, USA) an automatic pipette (Eppendorf, Warsaw, Poland). This was the same suspension that was applied to the surface of 30HGSA steel in subsequent research stages. Any excess suspension was spun off using a spin coater (POLOS Spin150i-NPP, SPS Putten, The Netherlands ) for 20 s at 1000 rpm. This specimen preparation of graphene oxide flakes suspended in the aqueous dispersion enabled imaging using a Scanning Transmission Electron Microscopy (STEM) (Quanta 250 FEG SEM, FEI, Hillsboro, OR, USA) detector coupled with a Quanta 250 FEG scanning electron microscope (Quanta 250 FEG SEM, FEI, Hillsboro, OR, USA). STEM imaging was performed under the following parameters: spot size 3, accelerating voltage (HV) 30 kV, and dwell time 10 µs.

A microstructure examination was performed on metallographic sections taken from cross-sections of flat 30HGSA steel samples. The sections were prepared using a cutting machine, an Opal 410 automatic embedding (ATM GmbH, Mammelzen, Germany) press and a Saphir disc grinder-polisher (ATM GmbH, Mammelzen, Germany). The microstructure of the samples was revealed by chemical etching with 5% Nital. Observation was carried out using a Zeiss SmartZoom 5 steerable microscope (Carl Zeiss Meditec AG, Göschwitzer Straße 12, 07745 Jena, Germany) at magnifications ranging from 100 to 1000 times. A PlanApo D 1.6/0.1 FWD 36 mm objective was used. Structural tests were performed on samples in the base material (BM), after shot peening (BM + SP), with a graphene oxide coating (BM + GO) and after hybrid graphening (BM + GO + SP). The microstructure of the surface layer of each of the above-mentioned sample types was analyzed by photographing the cross-sectional surfaces.

Surface analysis of 30HGSA steel in the base material (BM), with a deposited graphene oxide layer (BM + GO), after shot peening (BM + SP), and after hybrid graphening (BM + GO + SP), both before and after three-point bending, was carried out using a Quanta 250 FEG scanning electron microscope. In this case, backscattered electron imaging was performed using an ETD-BSE (Everhart–Thornley Detector with Backscattered Electron mode). The imaging parameters were accelerating voltage 10–20 kV, spot size 3.

The Quanta 250 FEG scanning electron microscope equipped with an EDAX detector was also used for elemental composition analysis of the sample surface in the base material, using a 30 kV accelerating voltage and spot size 5.

#### 2.3.2. Research Methodology for X-Ray Diffraction (XRD)

The crystallographic structure of steel samples was confirmed by means of X-ray studies. Diffraction measurements for the mentioned samples were carried out using the BRUKER D8 Discover diffractometer equipped with a CuK_α_ radiator (λK_α1_ = 1.54056 Å, λK_α2_ = 1.54443 Å, Siemens KFL CU 2 K, 40 kV voltage, and 40 mA current in operating mode), Göbel FGM2 mirror and 1D LYNXEYE detector (Bruker AXS Advanced X-ray Solutions SE, Karlsruhe, Germany). The Bragg–Brentano diffraction geometry was applied. The diffraction angle 2θ ranged from 2 to 145° with a step of 0.01° and acquisition time of 3 s per step. The DIFFRAC.SUITE EVA application (supported by diffractometer producer) was applied for data processing. The studies were carried out at 298 K in the temperature-stabilized Anton Paar HTK 1200 N chamber. Phase analysis was performed using Match! ver. 4.2 application, provided by Crystal Impact, with support of the COD database.

#### 2.3.3. Research Methodology for Microhardness 

The microhardness of the starting material (30HGSA steel) was measured after base material (BM), coating with graphene (BM + GO), shot peening (BM + SP), and after hybrid graphening (BM + GO + SP). Measurements were taken on cross-sections of flat samples deep into the material using the Vickers method with a load of 0.981 N (HV0.1). The material samples were then embedded in thermosetting resin, ground and polished. The hardness of the material was then measured using an FLC-50A hardness tester. Measurements were taken using a camera integrated into the hardness tester and software compatible with the tester. Measurements were taken from approximately 50 µm to 1.5 mm into the substrate material in order to check how microhardness changes after different surface treatments.

#### 2.3.4. Research Methodology for Surface Roughness 

In order to examine the surface development after GO deposition, shot peening, and hybrid graphening, surface roughness measurements were performed. These measurements were performed using a MicroProf 100 optical profilometer. Ten measurements were taken for each sample, both along and perpendicular to the rolling direction. The results were averaged.

#### 2.3.5. Research Methodology for Electrochemical Corrosion

Electrochemical corrosion behaviour of steel specimens, tested in the form of unmodified state, after shot peening and graphene oxide (GO) coating, was investigated a Metrohm Autolab PGSTAT 101 potentiostat operated via NOVA 2.1 software. Experiments were performed in a glass electrochemical chamber with a three-electrode setup, using a platinum rod (Ø = 1 mm) as the counter electrode, an Ag/AgCl (3 mol/L KCl) electrode as the reference, and the steel 30HGSA components as the working electrodes. The electrolyte used in all tests was a NaCl/HCl solution. For all steel components, long-term open circuit potential (OCP) monitoring was carried out prior to recording potentiodynamic polarization plots.

#### 2.3.6. Research Methodology for Ageing and Thermal Shock

Environmental tests were also conducted to accelerate the ageing process of the material and assess the impact of temperature shocks. The samples were conditioned using specialized equipment. The first stage involved climate shocks, in which the samples were exposed to a wide range of temperature changes, and the second stage involved exposure to UV radiation. The temperature shocks ranged from −60 °C to +60 °C for 120 h (30 min at elevated temperature and 30 min at reduced temperature), which was ensured in a T/60/V2 Weisstechnik thermal shock chamber (Weiss Umwelttechnik GmbH, Heuchelheim, Germany). The transition between the warm and cold environments took place within 6 s. The material ageing process using UV radiation was carried out in a QUV/SPRAY/RP accelerated ageing chamber. UV-A 340 lamps simulating daylight were used as the light source in the ageing chamber (for a period of 120 h). The temperature during irradiation was 60 °C (for 4 h) and the radiation intensity was 0.83 W/m^2^ (for 4 h).

The effects of temperature shocks and ageing tests were presented using a Zeiss SmartZoom 5 microscope.

#### 2.3.7. Research Methodology for Residual Stress 

The aim of residual stress analysis was to evaluate the stress values in 30HGSA steel after GO deposition (BM + GO), surface shot peening (BM + SP), and hybrid graphening (BM + GO + SP). Residual stresses with were measured using a Rigaku PSF-3M X-ray diffractometer. The following parameters were used: voltage—30 kV, current—8 mA, and counting time per measurement point—5 s.

#### 2.3.8. Research Methodology for Three-Point Bending 

Force measurement in the static three-point bending tests was carried out in accordance with ISO 7438:2016 [30] (International Organization for Standardization: Geneva, Switzerland, 2016). A Zwick Roell hydraulic pulsator was used to determine the maximum force required to bend the samples after specific surface treatments (BM + GO, BM + SP + GO and BM + SP). The span between the lower supports was 60 mm. A static force was applied vertically and symmetrically between the supports. The tests were performed under displacement-controlled conditions with a crosshead speed of 1 mm/s. The diameter of the support rollers was 10 mm.

Force–displacement curves (displacement of the loading punch) were determined for three-point bending of 30HGSA steel in the base material (BM), with a deposited graphene oxide layer (BM + GO), after shot peening (BM + SP), and after hybrid graphening (BM + GO + SP).

During the static tests conducted at a temperature of 20 °C, temperature distribution measurements were performed using a FLIR thermal imaging camera. The distance between the camera and the specimen was approximately 0.6 m, and the camera resolution was 640 × 320 pixels.

## 3. Research Results

### 3.1. Structural Analysis GO

**Figure 3:**
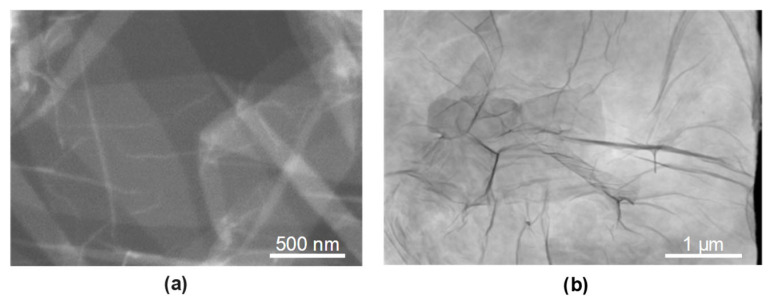
Structure of graphene oxide observed using a STEM detector coupled with a Quanta 250 FEG scanning electron microscope (**a**,**b**).

Transmission structural analysis of graphene oxide (GO) was performed using a Quanta 250 FEG scanning electron microscope equipped with a STEM detector. The observations revealed numerous overlapping graphene oxide flakes ranging in size from 3 to 150 µm (Figure 3a,b). These flakes, suspended in the dispersed aqueous solution, were deposited on the samples parallel to the surface during the GO deposition process.

### 3.2. Structural Analysis 30HGSA Steel

**Figure 4:**
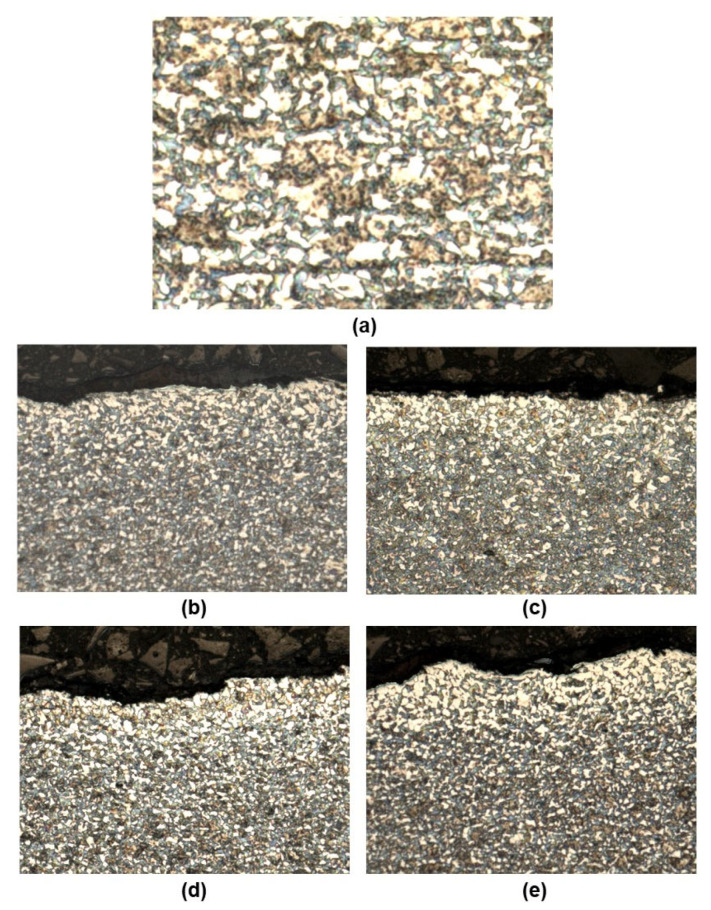
Surface of 30HGSA steel in the base material (BM) (**a**,**b**), after shot peening (BM + GO) (**c**), with a deposited graphene oxide layer (BM + SP) (**d**), and after hybrid graphening (BM + GO + SP) (**e**), obtained using a Zeiss SmartZoom 5 steerable microscope. Magnification: 1000× (**a**), 100× (**b**–**e**).

Structural tests of 30HGSA steel in its as-delivered state revealed the ferritic-pearlitic structure that is typical of this steel grade following heat treatment, with ferrite grains measuring 2 µm in size (see Figure 4a). Figure 4b–e show larger ferrite clusters with an increased grain size of up to 5 µm on the surface of the samples. These photographs depict the surface layer structure of the tested samples in their base material (BM) (Figure 4b), with a graphene oxide coating (BM + GO) (Figure 4c), after shot peening (BM + SP) (Figure 4d), and after hybrid graphening (BM + GO + SP) (Figure 4e). Figure 4d,e show the surface development and waviness resulting from the shot peening process, which increases roughness.

**Figure 5:**
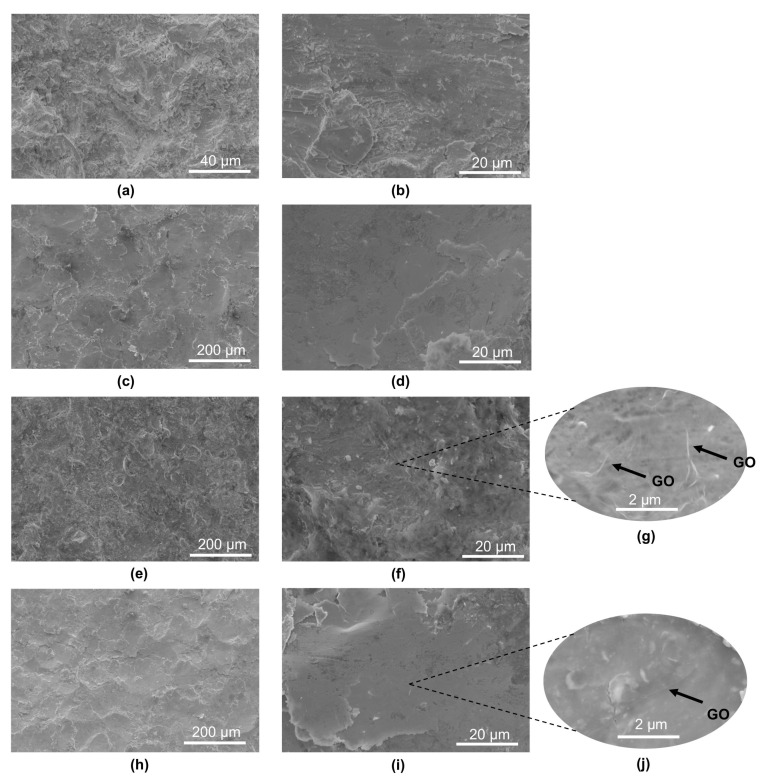
Surface topography of 30HGSA steel in the base material (BM) (**a**,**b**), after shot peening (BM + SP) (**c**,**d**), with a deposited graphene oxide layer (BM + GO) (**e**–**g**), and after hybrid graphening (BM + GO + SP) (**h**–**j**), obtained using a BSE detector coupled with a Quanta 250 FEG scanning electron microscope.

The topography of the tested base material samples in the base material (BM) (Figure 5a,b) revealed significant surface development, indicating that the 30HGSA steel sheets had undergone a sandblasting process during manufacturing. This is confirmed by the presence of 55.14 (±5.57) wt.% Si and 40.52 (±4.39) wt.% O, as determined using an EDAX detector coupled with the Quanta 250 FEG microscope. These elements are the main constituents of sand.

As mentioned, the base material (BM) exhibited considerable surface development resulting from the production process. However, the shot peening treatment further increased this surface development, which is confirmed by the surface roughness measurement results.

The surfaces of the BM + SP samples (Figure 5c,d) and BM + GO + SP samples (Figure 5h–j) exhibit typical features of the shot peening process, with visible indentations from the impacting shot particles, resulting in noticeable surface deformation (work hardening).

The presence of graphene oxide coating on the surfaces of the BM + GO (Figure 5e–g) and BM + GO + SP (Figure 5h–j) samples was confirmed using electron microscopy and is indicated in selected images (Figure 5g,i) by arrows. The graphene oxide coating in the SEM image formed a uniform layer of graphene oxide and was 20–40 nm thick (Figure 5g). It is more pronounced in the case of the GO coating deposited on the surface of the BM + GO (Figure 5g), sample, whereas after shot peening process, the GO flakes to hammer in the top layer, making it less visible (Figure 5j).

### 3.3. X-Ray Diffraction

**Figure 6:**
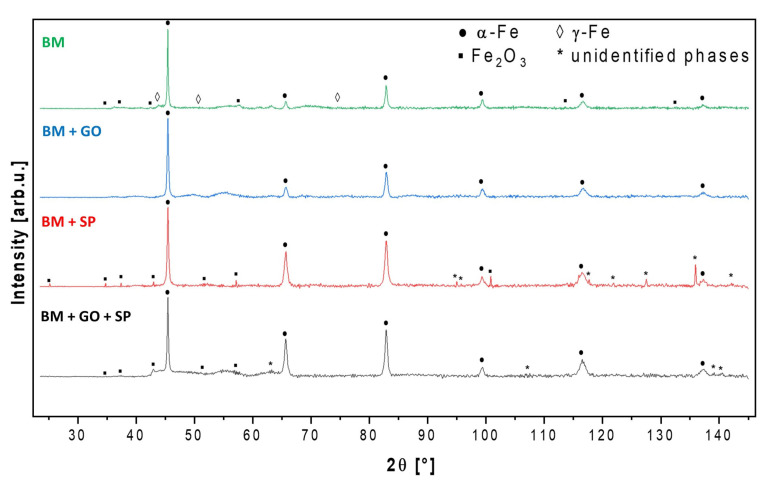
A complete set of XRD patterns of investigated steel samples along with phase analysis.

The results of XRD measurements and phase analysis are presented below in Figure 6. Due to the strong phosphorescence signal revealed by exposure of the ferrous materials to copper radiation source, a single X-ray diffraction measurement was conducted for a minimum of 12 h, followed by background subtraction and λK_α2_ component removal before the actual phase analysis. In the angular range 2θ from 2 to 25°, no diffraction signals characteristic for the tested samples were observed (including the ~10° region, where periodic carbon-like structures could be revealed), hence they were omitted from the presented XRD patterns. The base structure of all tested samples was based on typically ferritic steel (α-Fe), and in the case of the (BM) sample—with trace amounts of austenitic iron (γ-Fe).

Additionally, in the (SP) and (GO + SP) samples, diffraction signals from minor Fe_2_O_3_ and some unidentifiable phase(s) were also detected. The (BM + GO) steel sample proved to be the most phase-homogeneous. Evidently, the graphene treatment did not macroscopically affect the structure of the base material.

### 3.4. Microhardness Testing

**Figure 7:**
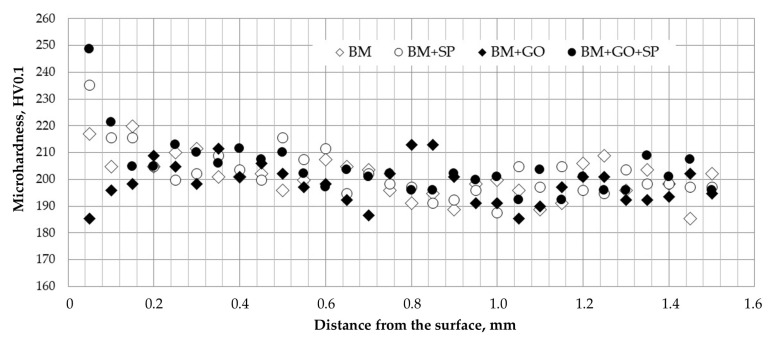
Microhardness distribution of 30HGSA steel deep into the material of samples with graphene oxide coating (BM + GO), after shot peening (BM + SP), after hybrid graphening (BM + GO + SP) and for materials in the BM delivery state.

Microhardness distribution tests were performed on flat samples of 3 mm-thick 30HGSA steel in its delivery condition. The results showed a slightly increased microhardness of approx. 220 HV0.1 in the surface layer at a depth of 50 µm, with a core microhardness of approx. 200 HV0.1 (see Figure 7). This increase was due to shot blasting of the sheet metal surfaces, which was carried out by the steel manufacturer. Depositing graphene oxide on the surface of the samples caused a slight decrease in microhardness in the surface layer to approximately 185 HV0.1. This was due to the effect of plasma cleaning when preparing the samples for coating with graphene oxide (BM + GO, Figure 7). Ball milling the sample material in its original state in accordance with this treatment increased the microhardness to approximately 235 HV0.1 at a depth of 50 µm (BM + SP, Figure 7). This increase is due to known processes occurring during ball milling, such as plastic deformation of the material and strengthening caused by surface crushing. Samples subjected to hybrid graphening treatment exhibited the most significant increase in microhardness in the surface layer, reaching approximately 250 HV0.1 at a depth of 50 µm, while maintaining consistent shot peening and graphene oxide coating parameters (BM + GO + SP, Figure 7). Microhardness increased by 25% compared to the base material. The observed changes in microhardness in the individual processes reached a depth of approximately 200 µm, with a downward trend for the (BM + GO + SP) and (BM + SP) samples, and an upward trend for the BM + GO sample, which reached a microhardness of approximately 200 HV0.1 in the base material.

### 3.5. Structural Roughness

The deposition of graphene oxide on the surface of 30HGSA steel resulted in a reduction in surface roughness. The graphene oxide flakes formed a relatively uniform layer that filled the irregularities created during the steel rolling process. As a result of GO deposition, a decrease in surface roughness parameters was observed for the BM and BM + GO + SP samples compared to BM and BM + SP, by approximately 2.0% (Ra) and 6.8% (Ra), respectively. In contrast, shot peening led to an increase in surface roughness by approximately 27.6% (Ra) (Table 2).

### 3.6. Residual Stresses

**Figure 8:**
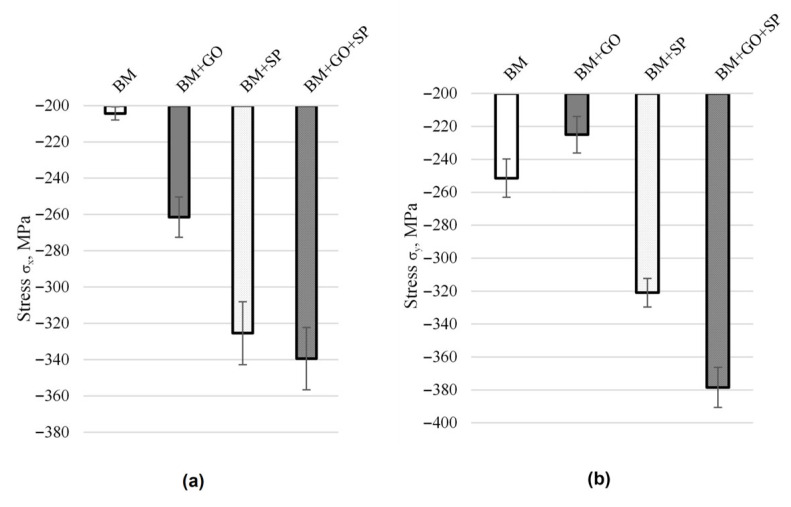
Transverse (σ_x_) (**a**) and longitudinal (σ_y_) (**b**) residual stresses in 30HGSA steel in the base material condition (BM), with a deposited graphene oxide layer (BM + GO), after shot peening (BM + SP), and after hybrid graphening (BM + GO + SP).

Residual stress analysis revealed that the base material in the base material exhibited a compressive stress state, resulting from surface enhancement treatments applied during the manufacturing process (Figure 8). Subsequently, due to the effects of low-temperature RF plasma treatment and graphene oxide deposition, partial stress relaxation occurred on the surface in terms of longitudinal stresses. The most favourable compressive stress state was observed in samples subjected to hybrid graphening (i.e., shot peening performed on a surface also coated with a graphene oxide layer).

### 3.7. Electrochemical Corrosion

**Figure 9:**
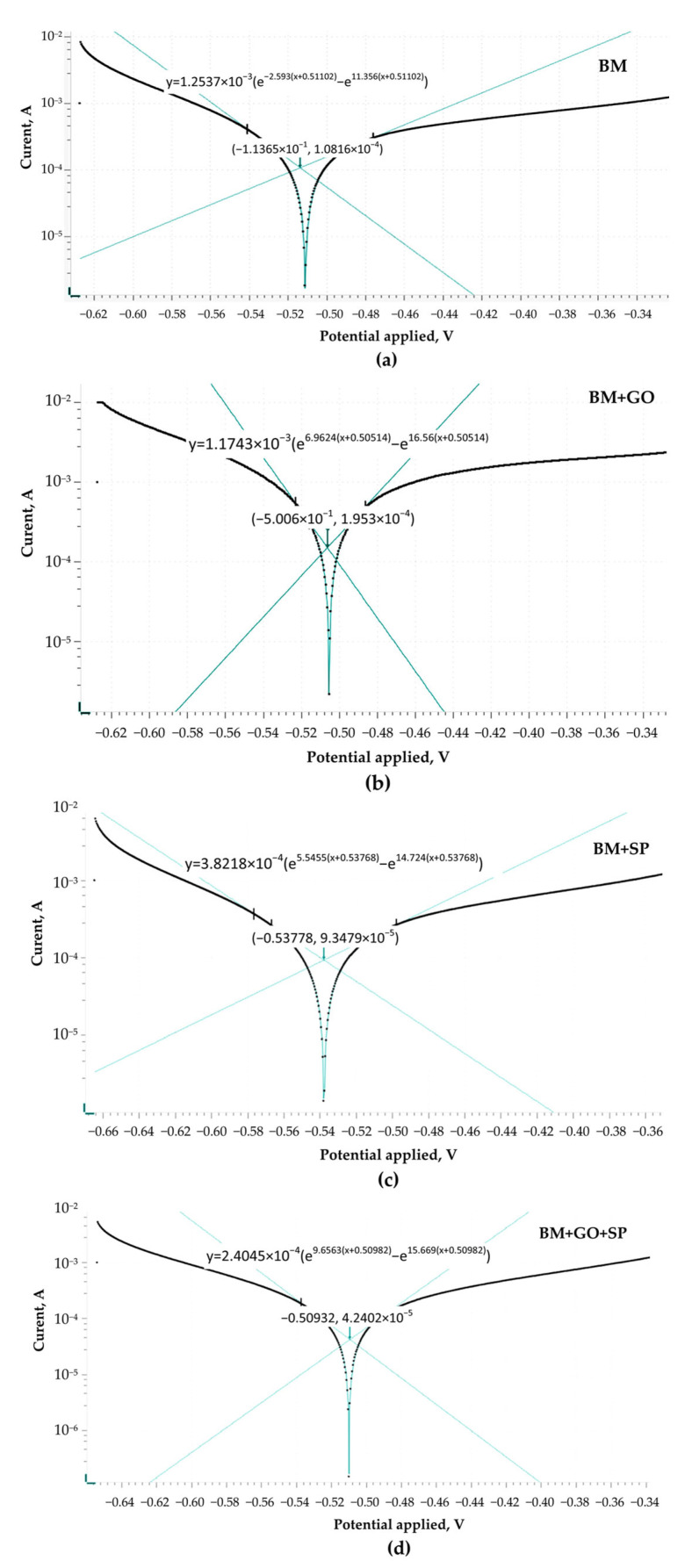
Evaluation of corrosion kinetics based on Tafel analysis of unmodified steel as a base material (BM) (**a**), steel covered by GO (BM + GO) (**b**), steel after shot-peening process (BM + SP) (**c**), hybrid graphening (BM + GO + SP) (**d**).

The electrochemical data demonstrate that the surface modification affects the corrosion behaviour of the 30HGSAsteel. The nonmodified steel acting as a base material (BM) (Figure 9a) exhibited the highest corrosion current density and corrosion rate, confirming its limited resistance in the tested NaCl/HCl environment. Application of graphene oxide (BM + GO) (Figure 9b) covering layer to the BM surface did not improve the corrosion performance. In contrast, shot peening (BM + SP) (Figure 9c) led to an improvement, manifested by a decrease in corrosion current density and an almost threefold reduction in the corrosion rate compared to BM, which can be attributed to surface hardening and compressive stresses introduced by the peening process. However, the most pronounced improvement, was observed after hybrid graphening (BM + SP + GO) (Figure 9d). This sample exhibited the lowest corrosion current density, the highest polarization resistance of 164.22 kΩ, and the lowest corrosion rate of 0.46241 mm per year among all tested materials (Table 3). The synergistic effect of shot peening and graphene oxide coating provided enhanced stability of the surface and minimized its electrochemical activity in aggressive chloride-containing environments.

### 3.8. Ageing and Thermal Shock Testing

**Figure 10:**
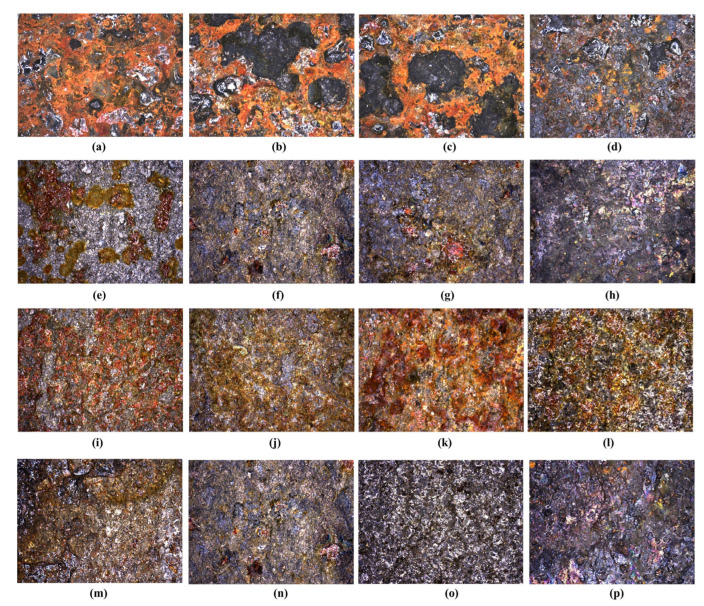
Surface topography of 30HGSA steel after ageing directly on surfaces in (BM) (**a**), (BM + SP) (**b**), (BM + GO) (**c**), (BM + SP + GO) (**d**) and on the opposite surface (BM) (**e**), (BM + SP) (**f**), (BM + GO) (**g**), (BM + SP + GO) (**h**). Surface topography of 30HGSA steel after exposure to thermal shocks on surfaces in (BM) (**i**), (BM + SP) (**j**), (BM + GO) (**k**), (BM + SP + GO) (**l**) and on the opposite surface (BM) (**m**), (BM + SP) (**n**), (BM + GO) (**o**), (BM + SP + GO) (**p**), obtained using Zeiss SmartZoom 5 steerable microscope with a magnification of ×100.3.9. Three-Point Bending Test.

Figure 10 shows the topography of the surface of 30HGSA steel after ageing and after exposure to thermal shocks. The analysis was performed using a Zeiss SmartZoom 5 steerable microscope mode at ×100 magnification. Both -surfaces directly subjected to treatment (ageing or thermal shocks) and the opposite surfaces in various material states: in the base material (BM), after shot peening (BM + SP), after coating with a GO layer (BM + GO) and after hybrid graphening (BM + SP + GO).

**Figure 11:**
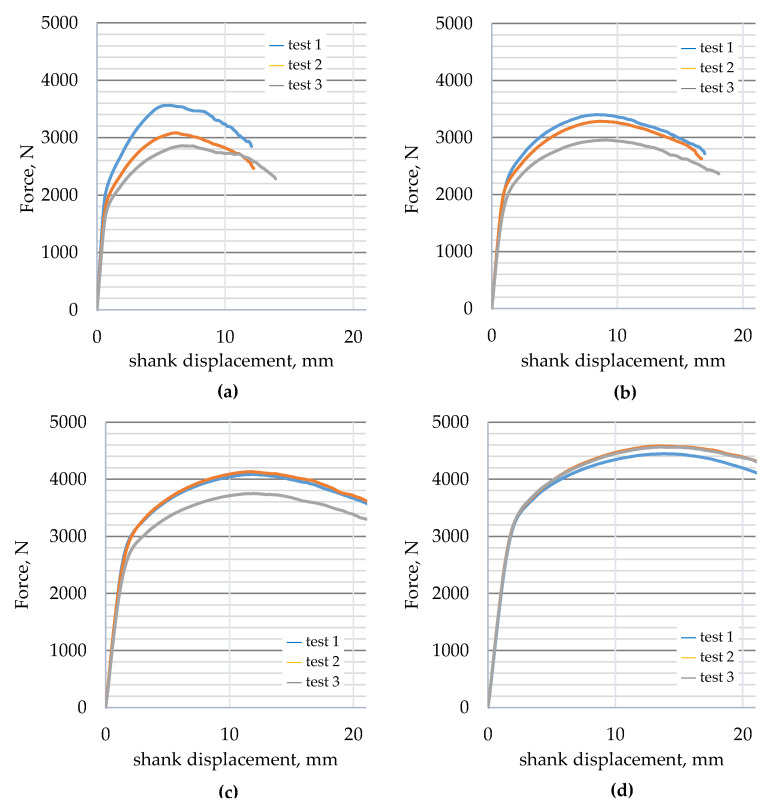
Force deflection curves for 30HGSA steel samples in the base material (BM) (**a**), with a deposited graphene oxide layer (BM + GO) (**b**), after shot peening (BM + SP) (**c**), and after hybrid graphening (BM + GO + SP) (**d**).

**Figure 12:**
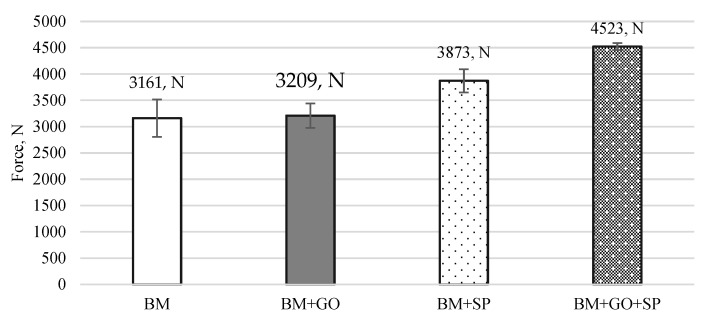
Comparison of maximum force in three-point bending of 30HGSA steel in the base material (BM), with a deposited graphene oxide layer (BM + GO), after shot peening (BM + SP), and after hybrid graphening (BM + GO + SP).

The results of the three-point bending tests on 30HGSA steel in its base material, with a deposited graphene oxide layer (BM + GO), after shot peening (BM + SP), and after hybrid graphening (Figure 11) were analyzed. Changes in force as a function of the displacement of the loading pin were determined for three test specimens. Samples in the BM delivery state (Figure 11a) and with a deposited graphene oxide layer (BM + GO), (Figure 11b) showed that depositing graphene oxide on the steel surface did not significantly affect changes in force as a function of displacement, as was observed with BM samples. Tests on samples after surface shot peening (BM + SP) (Figure 11c) showed an increase in maximum force of ~26% (Figure 12). The most favourable results of the three-point bending tests, however, were obtained after hybrid graphening (BM + GO + SP) (Figure 11d), with an increase in maximum force of approximately 43% compared to the base material BM. The test results were averaged from three tests (measurements).

**Figure 13:**
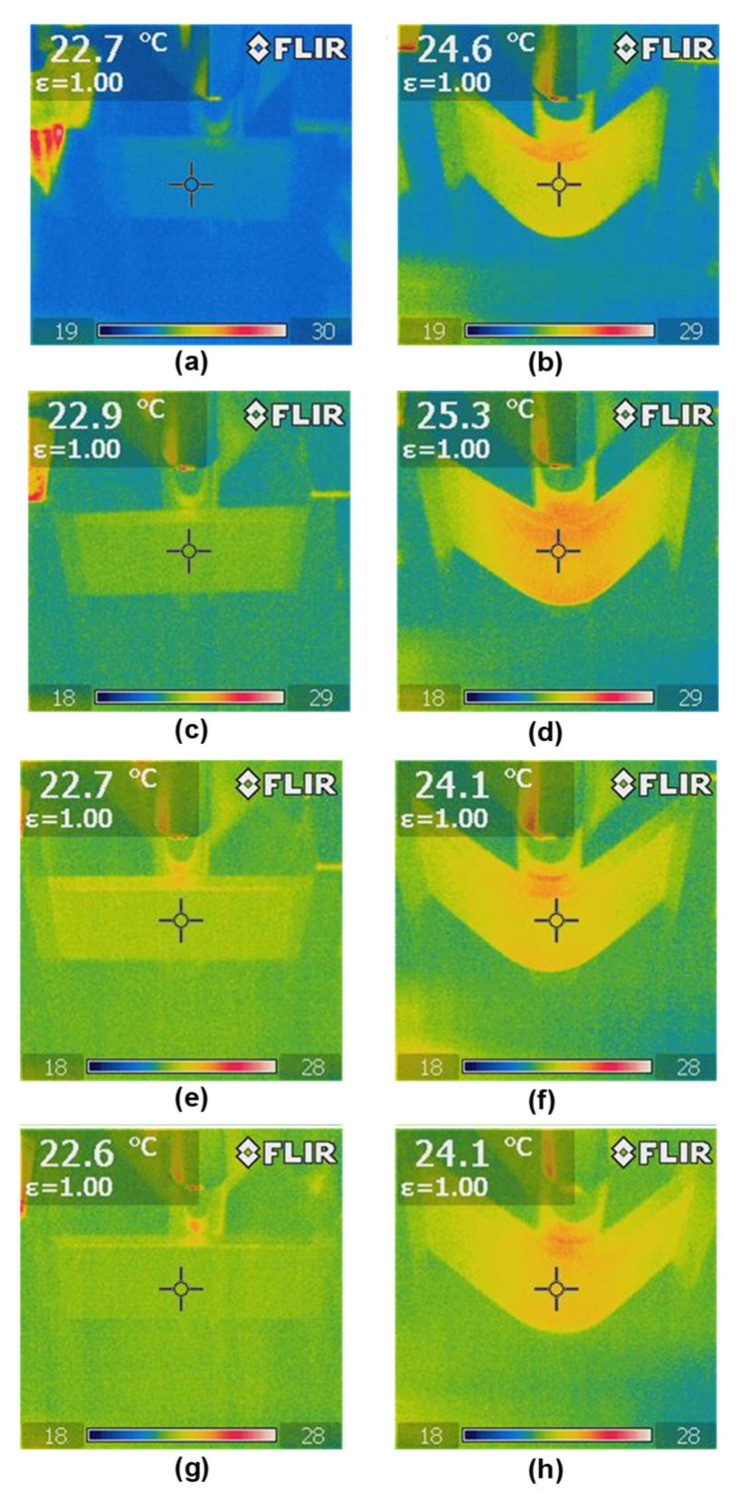
Selected thermal images of temperature distribution captured using a FLIR thermal imaging camera for 30HGSA steel in the base material (BM) (**a**,**b**), with a deposited graphene oxide layer (BM + GO) (**c**,**d**), after shot peening (BM + SP) (**e**,**f**), and after hybrid graphening (BM + GO + SP) (**g**,**h**), taken before (**a**,**c**,**e**,**g**) and at the final stage of the three-point bending tests (**b**,**d**,**f**,**h**).

Thermal imaging using a FLIR camera enabled the analysis of temperature changes before (Figure 13a,c,e,g) and at the final stage of the three-point bending tests (Figure 13b,d,f,h) for BM samples (Figure 13a,b), BM + GO (Figure 13c,d), BM + SP (Figure 13e,f), and BM + GO + SP (Figure 13g,h).

A slight increase in temperature was observed during the final stage of three-point bending of the samples, which was caused by internal friction in the plastically deformed material. The temperature of the samples varied slightly in the final stage of bending, resulting from the different levels of surface improvement.

**Figure 14:**
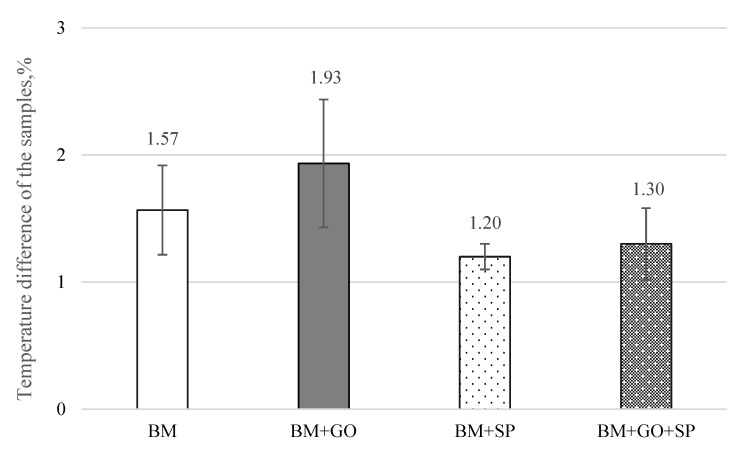
Temperature difference before and at the final stage of the three-point bending test for 30HGSA steel samples in the base material (BM), with a deposited graphene oxide layer (BM + GO), after shot peening (BM + SP), and after hybrid graphening (BM + GO + SP).

Uniform deposition of graphene oxide on the surface of 30HGSA steel (BM + GO) resulted in a 10% increase in surface temperature equivalent to 2.4 °C (average value from 3 measurements), measured using a FLIR thermal imaging camera during the three-point bending test (Figure 14).

The deposited graphene oxide layer reduces heat exchange with the environment, meaning the sample releases heat more slowly during the three-point bending process. The graphene flakes cracked and covered the substrate in the final test results, yet the temperature of the samples with the deposited graphene oxide layer could still be maintained at a higher level.

However, at the final stage of the test, graphene flakes began to fracture, disrupting the insulating barrier. Despite this, the temperature of the GO-coated samples remained elevated. The graphene oxide flakes adhered to the steel surface in a parallel orientation, thereby slowing heat dissipation due to their insulating nature [7].

**Figure 15:**
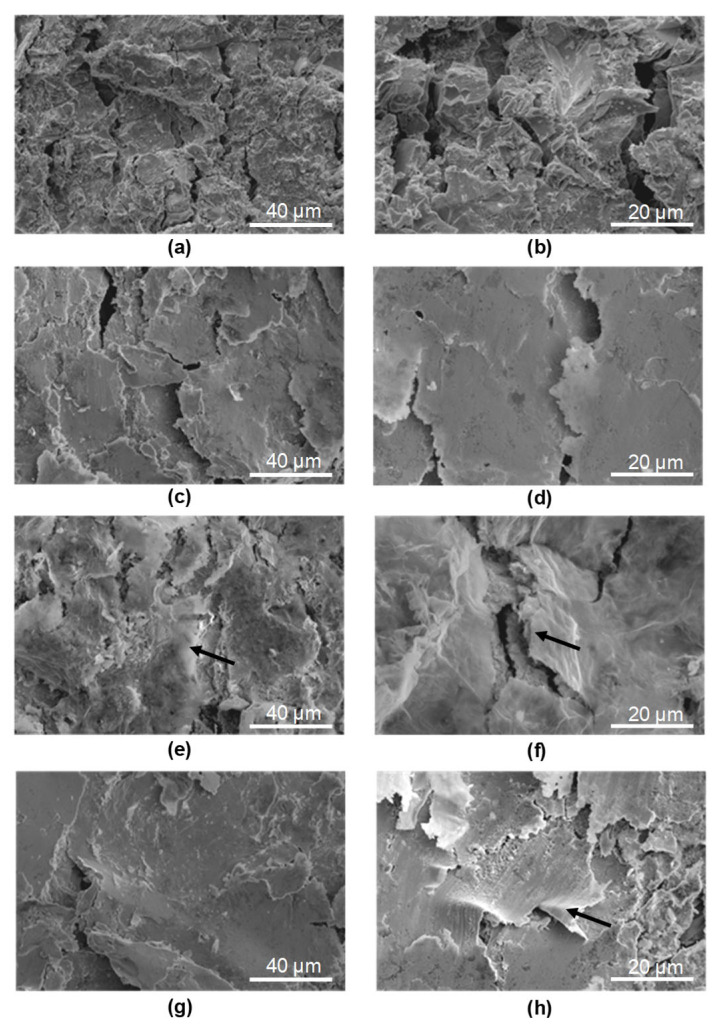
Surface topography of 30HGSA steel after three-point bending tests at the location of maximum deflection on the tensile side of the samples: in the base material (BM) (**a**,**b**), after shot peening (BM + SP) (**c**,**d**), with a deposited graphene oxide layer (BM + GO) (**e**,**f**), and after hybrid graphening (BM + GO + SP) (**g**,**h**), obtained using a BSE detector coupled with a Quanta 250 FEG scanning electron microscope.

In all tested samples BM (Figure 15a,b), BM + SP (Figure 15c,d), BM + GO (Figure 15e,f), and BM + GO + SP (Figure 15g,h) cracks perpendicular to the longitudinal edge of the sample appeared in the areas of maximum deflection. A network of parallel cracks was most visible in the base material (BM) (Figure 15a,b). During bending, graphene oxide flakes fractured, exposing the underlying 30HGSA steel substrate (Figure 15c,d) (indicated by arrows).

In the shot peening samples (BM + SP) (Figure 15c,d) and hybrid graphening samples (BM + GO + SP) (Figure 15g,h), the cracks were partially covered by the strengthened surface layer formed during shot peening.

## 4. Discussion

Analysis of the obtained test results confirmed the presence of graphene oxide flakes after their deposition on the sample surfaces. The results showed that graphene oxide deposition alone does not significantly improve the quality of the surface layer. However, the combination of graphene oxide deposition and shot peening leads to a noticeable enhancement in functional performance.

As a result of shot peening of the 30HGSA steel surface, both with and without the GO coating, surface deformation occurs within the plastically deformed top layer, generating compressive residual stresses. This surface deformation, characterized by an increase in compressive residual stresses, significantly contributes to the increase in the force required to bend the sample in three-point bending tests. The application of a graphene oxide coating alone (BM + GO), without subsequent shot peening, does not result in an increase in the bending force F [N] (Figure 12). Therefore, it is justified to introduce a beneficial compressive stress state and increase tensile strength through additional surface treatment in the form of shot peening [7].

In the case of hybrid graphening, i.e., shot peening of a surface coated with a graphene oxide layer exhibiting good adhesion to the substrate—the plastic deformation of the surface layer occurs with the participation of the GO coating.

**Figure 16:**
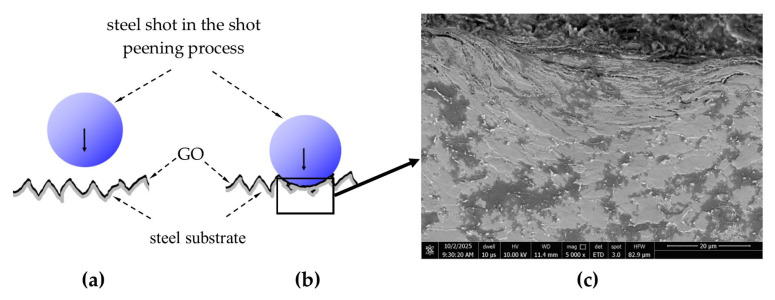
Interaction of shot particles with surface micro-irregularities during shot peening in subsequent stages: dynamic contact between the shot and the peening surface (**a**), elastic–plastic deformation of surface micro-irregularities after shot impact (**b**), plastic deformation of the steel surface layer (**c**).

The conducted research enabled the identification of the mechanism by which GO is introduced into the surface layer and demonstrated the resulting improvements in both mechanical and functional properties, as well as structural changes induced by the hybrid graphening process (Figure 16).

**Figure 17:**
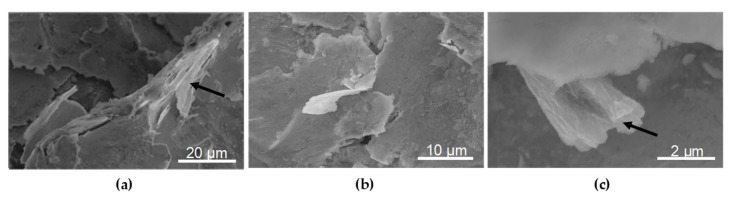
Graphene oxide (GO) flakes entrapped within the plastically deformed surface layer of 30HGSA steel after hybrid graphening (**a**–**c**).

The dynamic contact of the shot particle with surface micro-protrusions during the shot peening process leads to their plastic deformation along with the graphene oxide coating. This results in the entrapment of graphene oxide within the plastically deformed micro-irregularities (Figure 17a,b).

Graphene oxide trapped within the closed micro-irregularities behaves similarly to the bulk material under external loading, partially transferring its superior mechanical properties and thereby contributing to the increased strength of the tested samples.

This effect is illustrated in the images (Figure 17a,b), which show graphene oxide flakes entrapped beneath plastically deformed surface micro-irregularities (indicated by arrows) (Figure 17a), as well as the closure of micro-irregularities (indicated by arrows) (Figure 17b).

Tests on 30HGSA steel revealed a ferritic-pearlitic structure typical of the heat treatment process, which in this case was hardening and high tempering. It was observed that the deposition of graphene oxide on the surface results in a thin, uniform layer that adheres to the surface but may detach during use. To prevent this, the steel surface was pre-activated with RF plasma. However, the use of shot peening treatment on a surface with a previously applied graphene oxide layer additionally increases compressive stresses, develops the surface through increased roughness and microhardness, and improves corrosion resistance, which improves the functional properties.

The increase in roughness of the BM + SP and BM + GO + SP shot peened samples, expressed by the Rz roughness parameter of 62.5 and 61.8 µm, respectively, resulted in greater surface development and thus an increase in the heat dissipation area, resulting in a reduction in the temperature difference in these samples by 62 and 67% compared to BM + GO graphene oxide layer samples with a roughness Rz of 44.6 µm. It is also worth noting that the graphene oxide layer evenly covering the surfaces of the samples evens out the unevenness compared to the surfaces of the BM samples as delivered, for which Rz is 52.4 µm (Table 2). 

The current laboratory tests were performed on standardized sample fragments; therefore, their practical application requires further research. While the preparation of large-scale fragments should not be a problem, as it involves the use of larger chambers for plasma treatment and shot peening, the process of joining steel after hybrid graphene coating has not yet been tested. This is a new area of research, and the presented publication is only the beginning.

## 5. Conclusions

- Structural studies using an optical microscope and XRD revealed the presence of ferrite (α-Fe) and trace amounts of austenitic steel (γ-Fe).
- The surface of the base material (30HGSA steel), despite having undergone quenching and tempering, was also subjected to sandblasting. This was confirmed by surface elemental analysis of the samples. The sandblasting process was not indicated in the material specification provided by the steel manufacturer.
- The surface roughness of the base material samples was Ra = 9.05 µm. It slightly decreased after the GO coating process to Ra = 8.87 µm and increased after shot peening to Ra = 12.40 µm for BM + SP samples and Ra = 11.55 µm for BM + GO + SP samples.
- Shot peening and hybrid graphening resulted in an increase in compressive residual stresses. For BM + SP samples, the increase was σx by 158% and σy by 165%; for BM + GO + SP samples, the increase was σx by 127% and σy by 152%, relative to the stress state in the base material.
- The maximum force required for three-point bending increased by 43% after hybrid graphening for BM + GO + SP samples compared to the base material (BM).
- Surface topography analysis of BM + GO samples revealed uniform and durable graphene oxide coverage, which contributed to the reduction in surface roughness as a result of plasma cleaning prior to deposition.
- Examination of hybrid graphening samples (BM + GO + SP) after three-point bending revealed the presence of graphene oxide flakes embedded within the shot peening surface layer at the points of maximum deflection.
- Based on the conducted observations of the effects of GO and shot peening, a mechanism for hybrid graphening was proposed [7]. This mechanism is based on the entrapment of GO flakes within plastically deformed surface irregularities created by the impact of shot particles during the peening process.
- The surface images after thermal shocks and ageing confirm that the application of the SP + GO combination enhances the material’s resistance to environmental conditions acting on its surface.

## Figures and Tables

**Table 1:** Chemical composition of 30HGSA steel as specified in the delivery certificate (quality certificates No. 3966-16b).

Elemental Composition	C	Mn	P	S	Cr	Ni	Cu
[%]	0.31	0.99	0.017	0.0007	0.87	0.24	0.19

**Table 2:** Surface roughness of 30HGSA steel in the base material (BM), with a deposited graphene oxide layer (BM + GO), after shot peening (BM + SP), and after hybrid graphening (BM + GO + SP). Ra—arithmetic average roughness, Rq—root mean square roughness, Rz—average maximum height of the roughness profile (mean from five sampling lengths), Rp—maximum profile peak height above the mean line, Rv—maximum valley depth below the mean line, Rt—total height of the roughness profile. The results of the surface roughness measurements are presented in Table 2 at a 95% confidence level.

	Ra	Rq	Rz	Rp	Rv	Rt
	µm					
Surface of the base material (BM)	9.053	11.464	52.461	34.775	33.068	67.843
Surface of the GO-coated sample (BM + GO)	8.871	11.436	44.826	30.740	41.223	71.963
Surface of the shot peening sample (BM + SP)	12.406	15.542	62.514	32.894	48.133	81.027
Surface of the hybrid graphening sample (BM + GO + SP)	11.556	14.094	61.884	32.387	39.850	72.237

**Table 3:** The kinetic data derived from Tafel.

Sample	Ecorr. (V)	jcorr. (×10^−4^ A/cm^2^)	Polarization Resistance (kΩ)	Corrosion Rate (mm/Year)
BM	−0.51365	1.0361	91.019	2.4079
BM + SP	−0.53778	0.38998	129.09	0.90746
BM + GO	−0.50606	0.9705	36.202	2.2583
BM + GO + SP	−0.50932	0.19872	164.22	0.46241

## Data Availability

The raw data supporting the conclusions of this article will be made available by the authors on request.
